# Supplementary figures and images for: Quantitative proteomics reveals the selectivity of ubiquitin-binding autophagy receptors in the turnover of damaged lysosomes by lysophagy
Source: eLife. 2021 Sep 29;10:e72328. doi: 10.7554/eLife.72328 (PMC8523161; doi:10.7554/eLife.72328)

FIGURE 1G

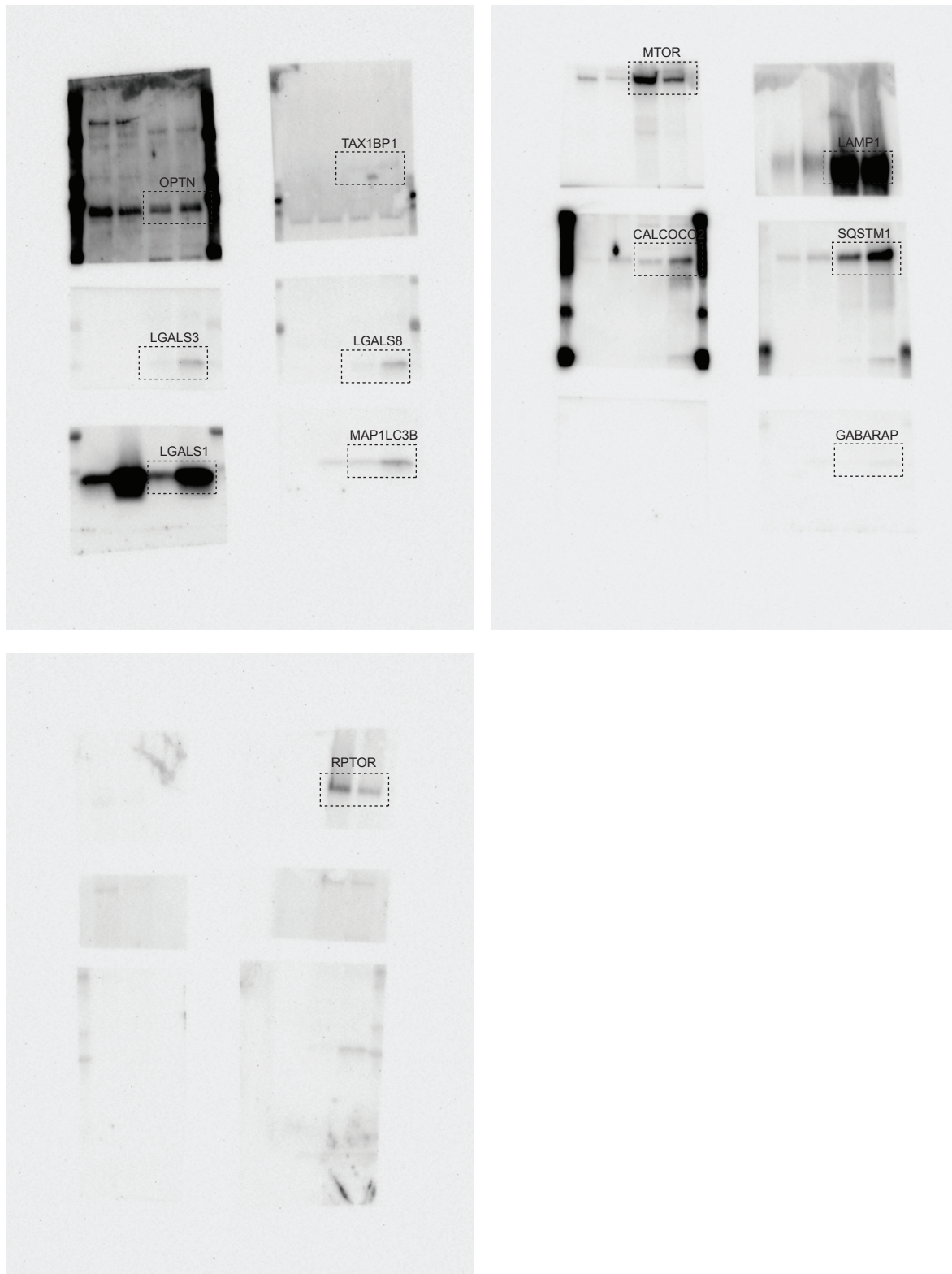

Supplement: Figure 1—source data 1. [file elife-72328-fig1-data1.pdf]

FIGURE S1B / FIGURE S1D / FIGURE S1H

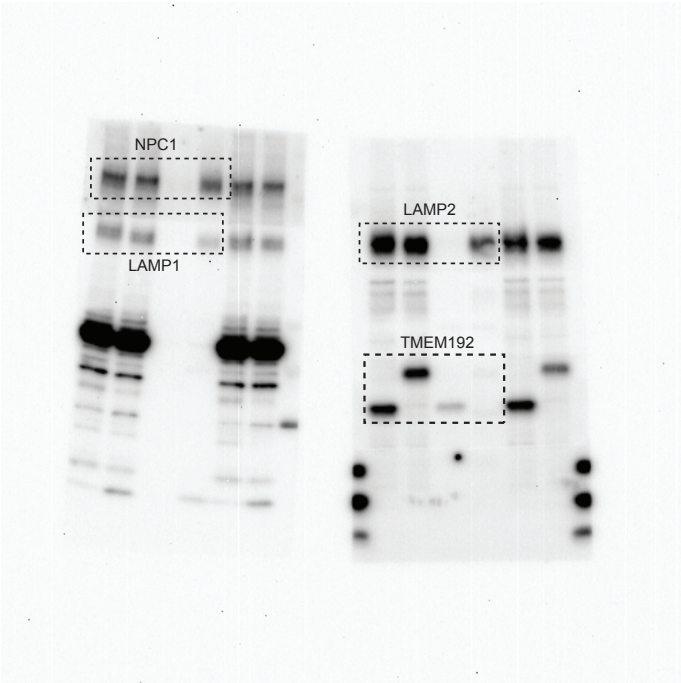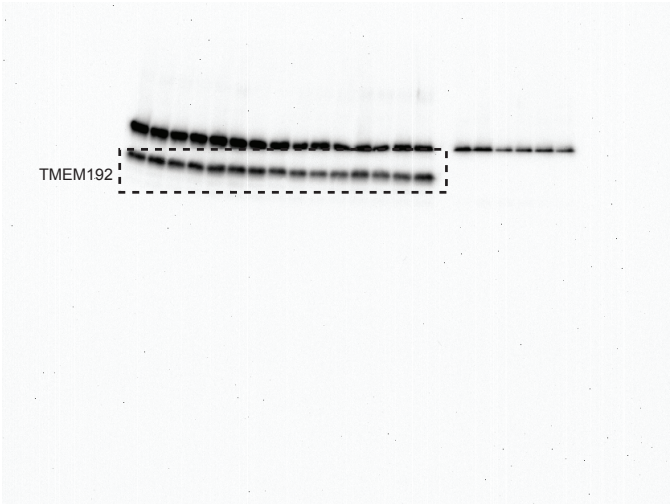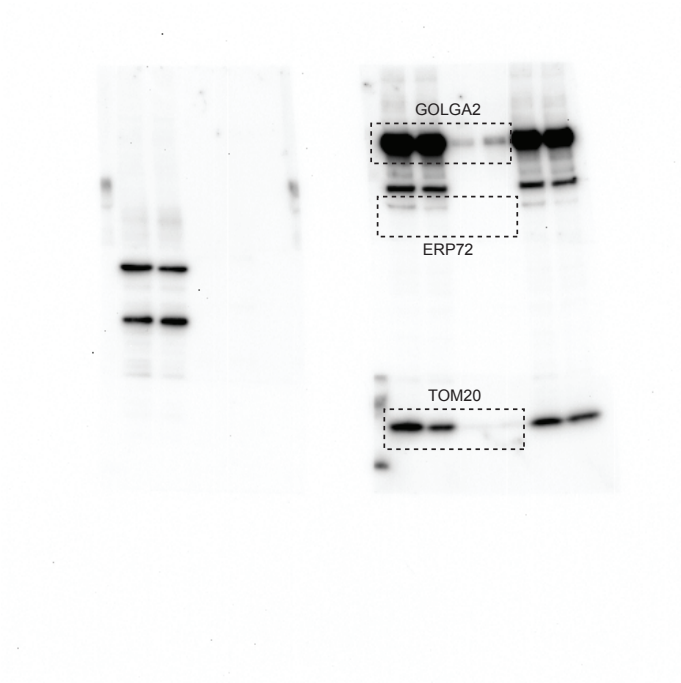

Supplement: Figure 1—figure supplement 1—source data 1. [file elife-72328-fig1-figsupp1-data1.pdf]

FIGURE S2A / FIGURE S3A / FIGURE S4B

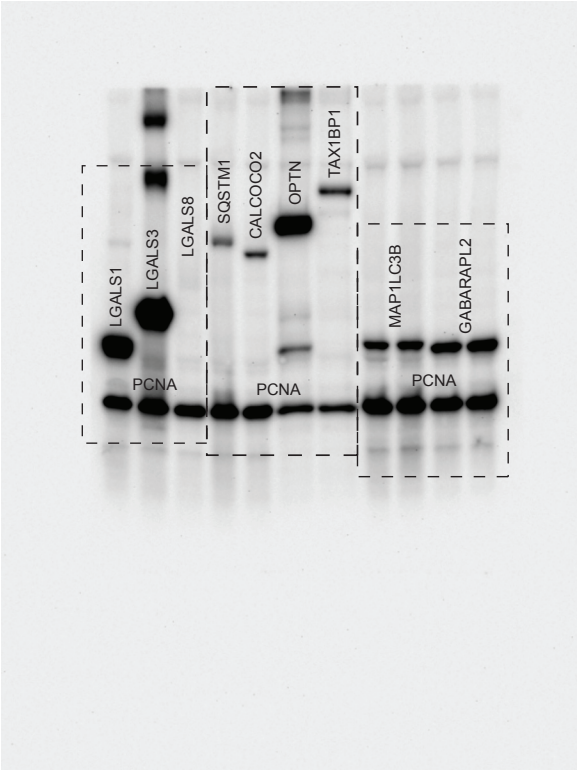

Supplement: Figure 2—figure supplement 1—source data 1. [file elife-72328-fig2-figsupp1-data1.pdf]

FIGURE S2A / FIGURE S3A / FIGURE S4B

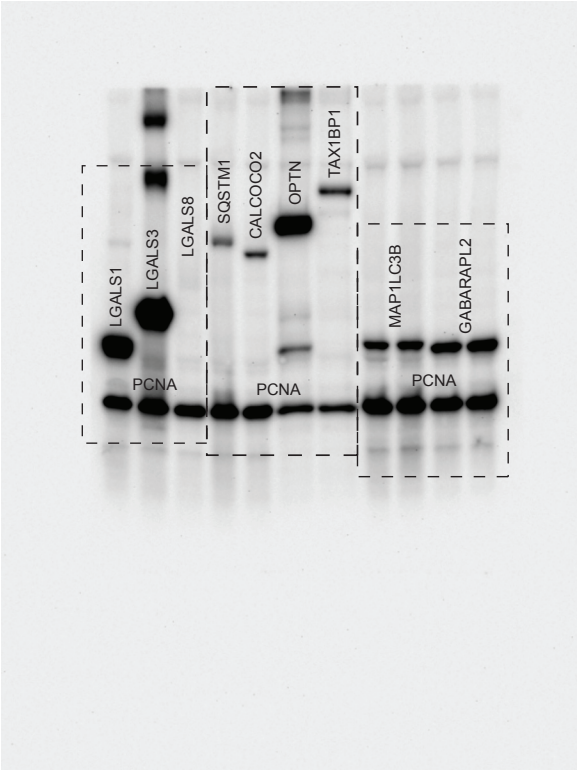

Supplement: Figure 3—figure supplement 1—source data 1. [file elife-72328-fig3-figsupp1-data1.pdf]

FIGURE S2A / FIGURE S3A / FIGURE S4B

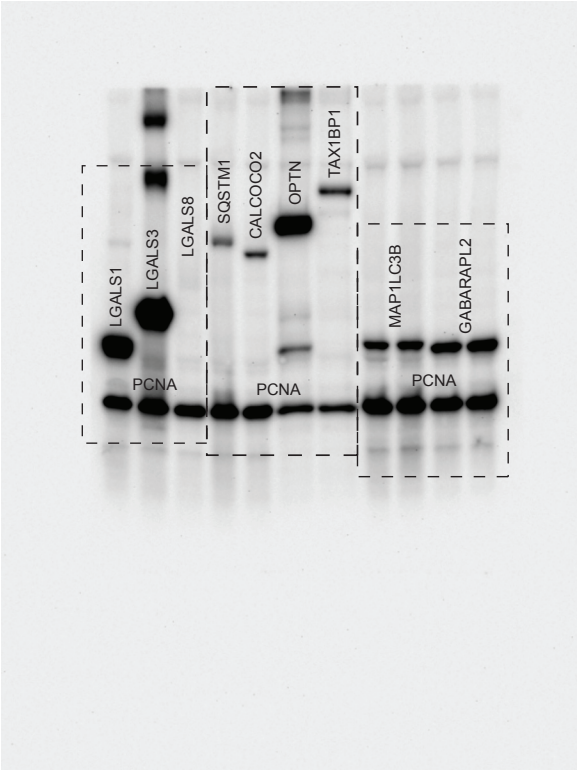

Supplement: Figure 4—figure supplement 1—source data 1. [file elife-72328-fig4-figsupp1-data1.pdf]

Figure 5 - source data 4. Uncropped blots for panel H.

5F  
Keima

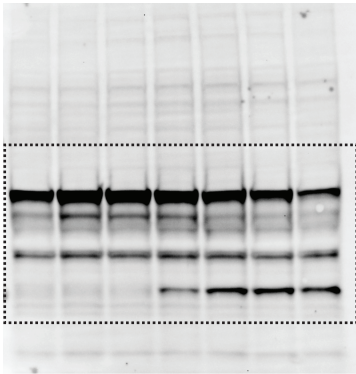

5H  
Keima

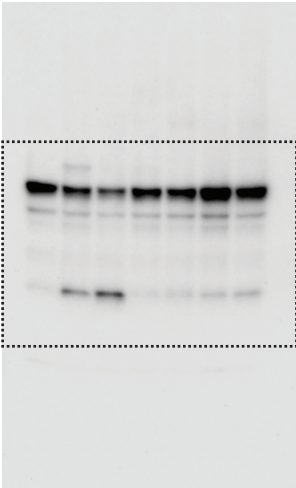

5F  
pTbk1

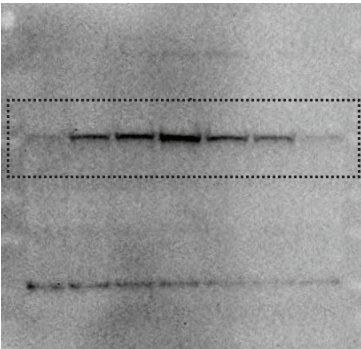

5H  
p-Tbk1

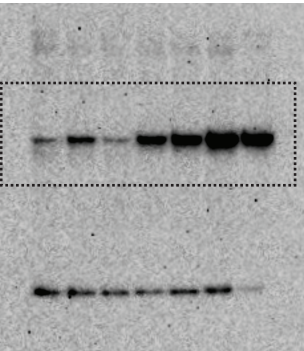

5F  
Tbk1

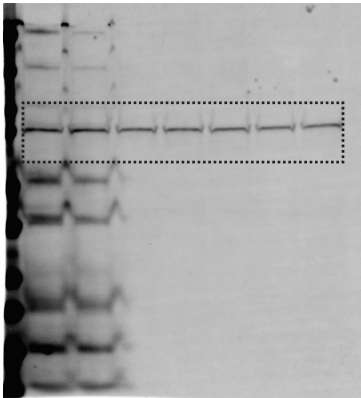

5H  
Tbk1

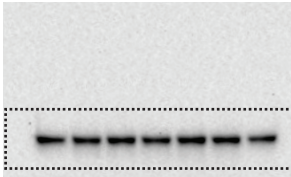

5H  
Actin

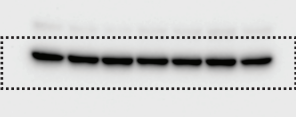

Supplement: Figure 5—source data 4. [file elife-72328-fig5-data4.pdf]

Figure 5-figure supplement 1B-source data 1

S5B  
Keima

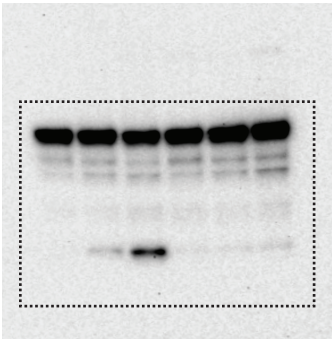

S5B  
p-Tbk1

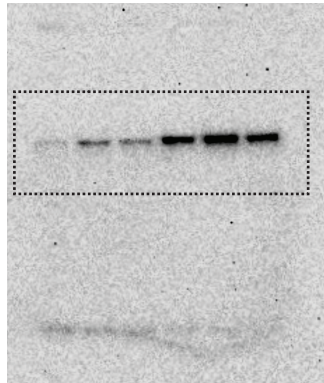

S5B  
FK2 (ub)

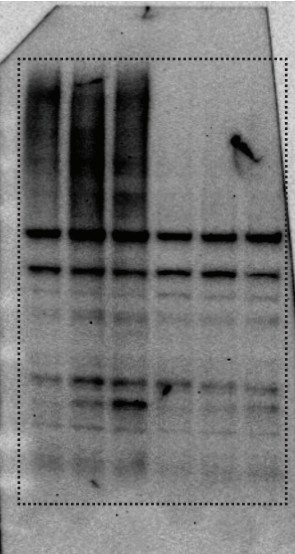

S5B  
Tbk1

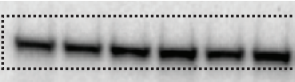

S5B  
FK2 (Actin)

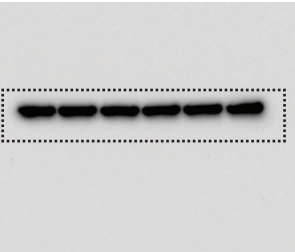

Supplement: Figure 5—figure supplement 1—source data 1. [file elife-72328-fig5-figsupp1-data1.pdf]

Figure 6-source data 5. Uncropped blots for panel 6C.

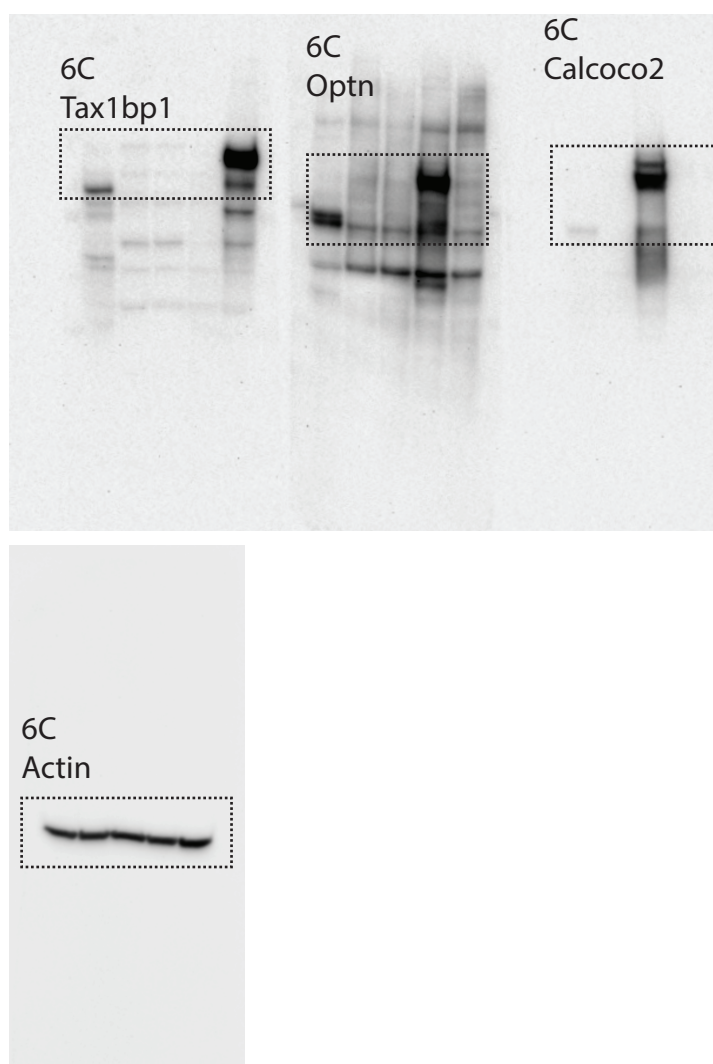

Supplement: Figure 6—source data 5. [file elife-72328-fig6-data5.pdf]

Figure 6-figure supplement 1A-F-source data 1. Uncropped blots.

S6A-F

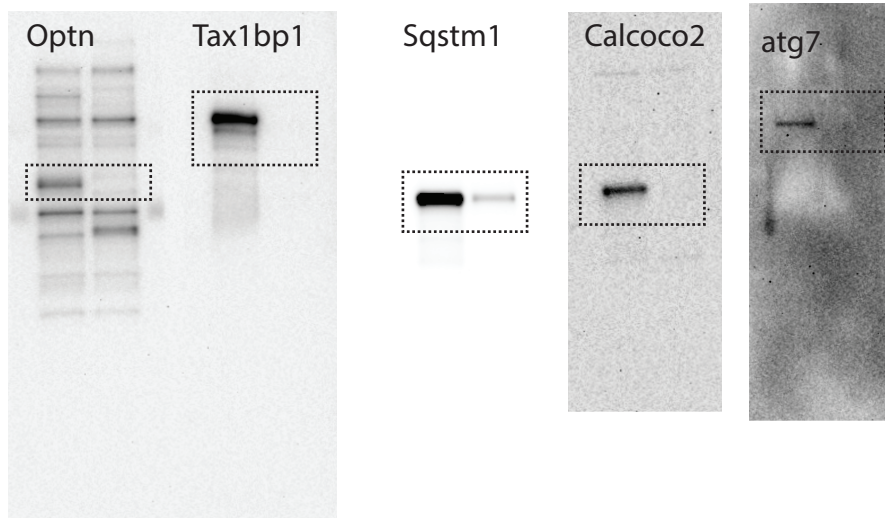

Actin  
Corresponds to

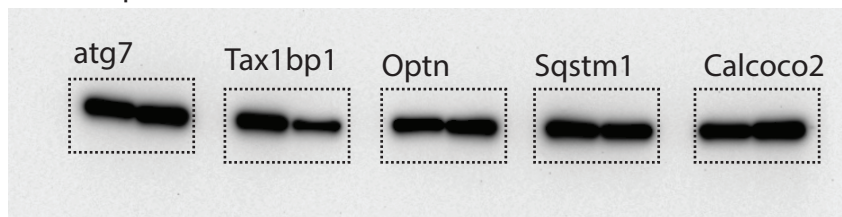

Supplement: Figure 6—figure supplement 1—source data 1. [file elife-72328-fig6-figsupp1-data1.pdf]

Figure S7. Analysis of LGALS3R186S recruitment to damaged lysosomes in iNeurons.

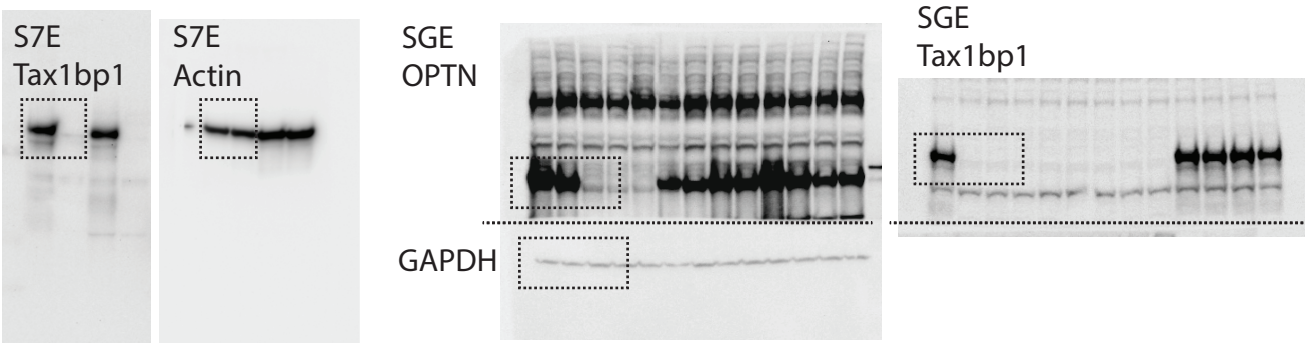

Supplement: Figure 7—figure supplement 1—source data 2. [file elife-72328-fig7-figsupp1-data2.pdf]

Figure 8. Structure-function analysis of TAX1BP1 and OPTN for lysophagy.

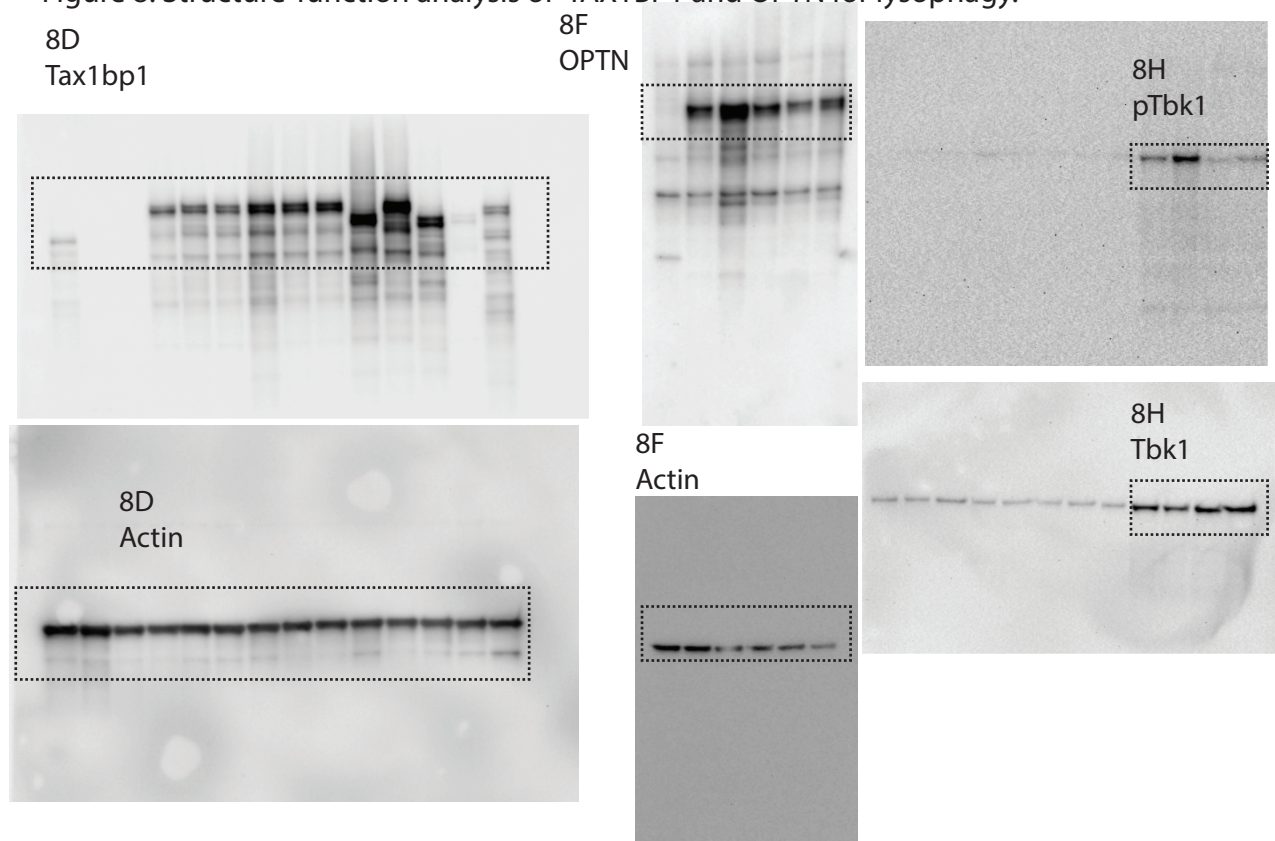

Figure 8D

pTBK1

cut  
GAPDH

TBK1

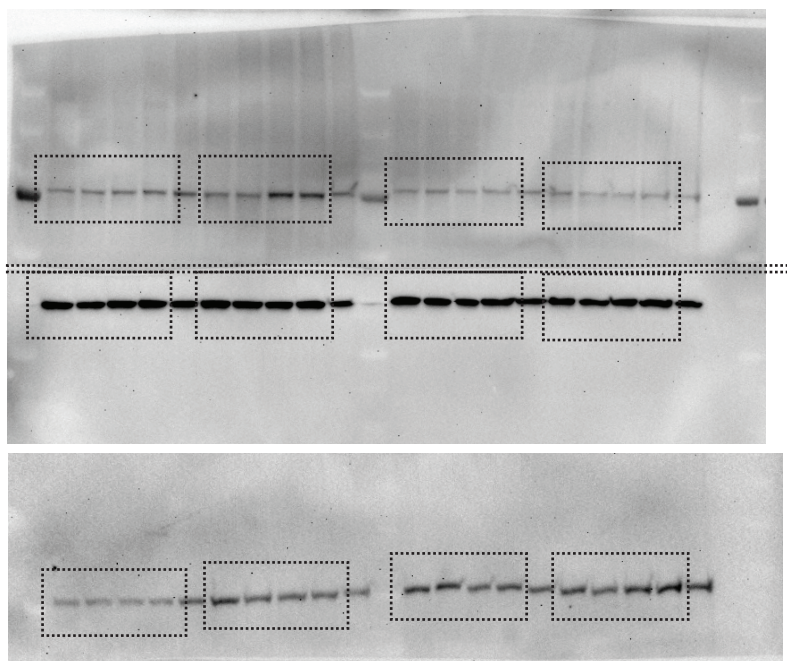

Supplement: Figure 8—source data 3. [file elife-72328-fig8-data3.pdf]
